# Supplementary material for: Characteristics of SARS-CoV-2 testing for rapid diagnosis of COVID-19 during the initial stages of a global pandemic
Source: PLoS One. 2021 Jul 9;16(7):e0253941. doi: 10.1371/journal.pone.0253941 (PMC8270193; doi:10.1371/journal.pone.0253941)
Supplement: S1 File — (PDF) [file pone.0253941.s001.pdf]

SUPPLEMENTAL MATERIALS

Table of Contents

S1 Fig. Key dates related to SARS-CoV-2 testing in Ontario (ON) over the study period 2

S1 Table. Characteristics of the 118 individuals with invalid test results 3

SI APPENDIX. Molecular-based Testing Supplementary 4

A.I.I. Supplementary Methods 4

S2 Table. Primer and probe sequences 6

A.I.II. Supplementary Test Performance Characteristics 7

S3 Table. Number of specimens tested by a single assay and with an additional assay(s) 7

S4 Table. Number of specimens by discordant result 7

S5 Table. Number of specimens by rRT-PCR assay result 8

S2 Fig. Scatter plot of cycle threshold (C_t) values for tests with multiple targets. 9

S6 Table. Relative difference in cycle 9

A.I.III. Supplementary Molecular-based Testing Summary 10

SII APPENDIX. Specimen-based Testing Supplementary 10

S7 Table. Summary for the number of specimens per individual 10

S8 Table. Number of same-day multi-specimen testing episodes categorized 11

S9 Table. Number and proportion of individuals by symptom status at time of testing 12

S3 Fig. Molecular-based test results 13

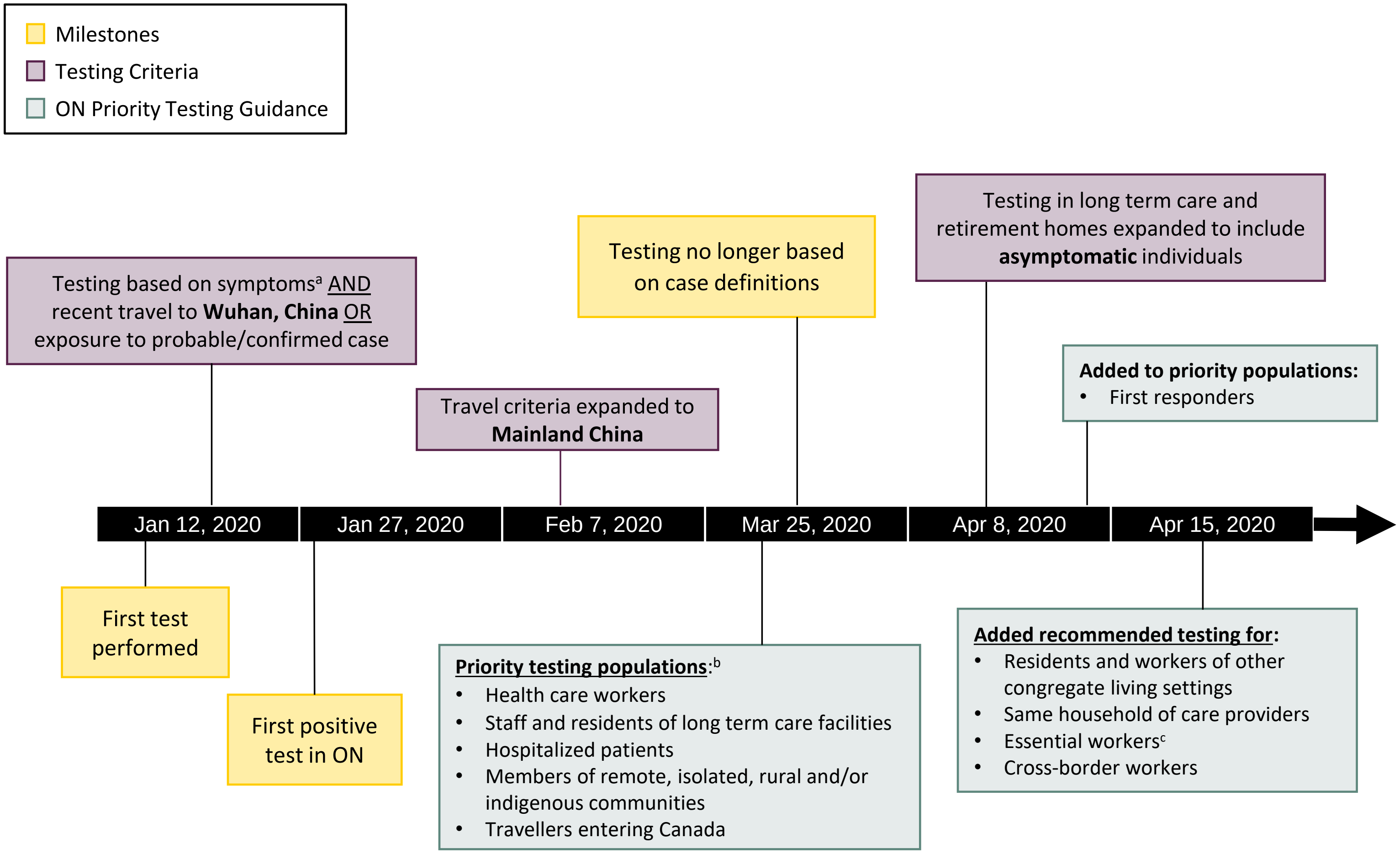

a. Fever and/or cough or difficulty breathing
b. Testing recommended if symptomatic (one of: fever, new/worsening symptom, clinical or radiologic evidence of pneumonia)
c. Provincial workers considered critical to preserving life, health and basic societal functioning - https://www.ontario.ca/page/list-essential-workplaces

S1 Fig. Key dates related to SARS-CoV-2 testing in Ontario (ON) over the study period.

S1 Table. Characteristics of the 118 individuals with invalid test results that did not have other specimens submitted to Public Health Ontario Laboratory, January 11, 2020 – April 22, 2020 with valid test results (positive, *n*=4; negative, *n*=23).

Characteristics	Test[a] Result Invalid (*n*=118)
Age, y—median (IQR)	44 (31–61)
Age group, y—No. (%)	
<1	0 (0.0)
1–4	2 (1.7)
5–19	8 (6.8)
20–39	34 (28.8)
40–59	38 (32.2)
60–69	14 (11.9)
70–79	10 (8.5)
80–89	5 (4.2)
≥90	6 (5.1)
Not specified	1 (0.8)
Gender—No. (%)[b]	
Male	53 (44.9)
Female	64 (54.2)
Other/Not specified	1 (0.8)
Reason for Testing[c]	
Symptoms[d]—No. (%)	
Yes	106 (89.8)
No	2 (1.7)
Not specified	10 (8.5)
Travel history[e]—No. (%)	
Yes	16 (13.6)
None reported	102 (86.4)
Exposure history[f]—No. (%)	
Yes	3 (2.5)
None reported	115 (97.5)
Healthcare worker—No. (%)	3 (2.5)
Ever hospitalized—No. (%)	10 (8.5)
Outbreak associated—No. (%)	4 (3.4)
Deceased (autopsy specimen)—No. (%)	0 (0.0)

[a]*Molecular-based testing included one or more of the following: end-point PCR + Sanger sequencing of the RNA-dependent RNA polymerase (RdRp) gene, rRT-PCR to detect the envelope (E) gene, RdRp gene and/or ORF1ab.*

[b]*Percentages have been rounded and may not total 100%.*

[c]*Reasons for testing were not mutually exclusive.*

[d]*Symptoms— Self-reported response or clinical assessment of fever, cough, sore throat, pneumonia, or other COVID-19 symptoms*

[e]*Travel history—Self-reported response to 'Travelled outside the country in the last 14 days?'.*

[f]*Exposure history—Self-reported response to 'Exposure to probable, or confirmed case?'.*

SI APPENDIX. Molecular-based Testing Supplementary

A.I.I. Supplementary Methods

RNA extraction and molecular-based assays

Automated processes were used for RNA extraction and included the NucliSENS easyMAG extractor (bioMérieux, QC, Canada), the *m*2000 platform (Abbott Inc., IL, USA), and the cobas® 8800 (Roche Diagnostics, Germany). All were used according to manufacturer instructions. For the Roche assay, a 400 µl aliquot of specimen was added to 200 µl of cobas® Omni Lysis Reagent (43% guanidine thiocyanate w/v). The instrument was loaded with a 400 µl aliquot of the above 600 µl combination of specimen/lysis reagent for testing.

The initial test for SARS-CoV-2 used easyMAG extracted RNA which was subjected to a nested end-point PCR[1] targeting a 192 base pair fragment of the SARS-CoV-2 RdRp gene, followed by Sanger sequencing using standard methods. Briefly, 5 µL extracted RNA was added to 20 µL of a reaction mixture containing 12.5 µL 2x Superscript-III One-step RT-PCR Master Mix, 1 µL BSA solution, $MgSO_4$ solution (5 nM), 1 µL Superscript-III RT enzyme solution, 3.1 µL of RNAse free and 1 µL each of 10 µM primers RdRp-PHL-WH-F (5'-TGCCATTAGTGCAAAGAATAGAGC-3') and RdRp-PHL-WH-R (5'-GCATGGCTCTATCACATTTAGG-3'). One-step RT-PCR amplification consisted of a pre-incubation at 50°C for 20 min and 94°C for 3 min, followed by initial denaturation at 95°C for 3 min, 45 cycles of denaturation at 95°C for 15 sec, annealing at 56°C for 15 sec, extension at 72°C for 30 sec and final extension at 72°C for 2 min. Next, 5 µL of the outer PCR product was added to 20 µL of a reaction mixture containing 12.5 µL Qiagen Taq PCR Master Mix, 3.1 µL of RNAse free and 0.4 µL each of 10 µM primers RdRp-PHL- WH-COV-3Fseq (5'-GCACCGTAGCTGGTGTCTCT-3'), and RdRp-PHL-WH-COV-3Rseq (5'-AATCCCAACCCATAAGGTGA-3'). Amplification of a 192 bp fragment was performed using the following cycling conditions: 95°C 3 min, 45 cycles at 95°C 30 sec, 56°C 30 sec, 72°C 30 sec, and a final extension at 72°C 10 min. The PCR product was purified sequenced using BigDye Terminator v3.1 Cycle Sequencing Kit (Applied Biosystems, Foster City) according to the manufacturer's instructions. Trimmed and cleaned sequencing results were aligned to the reference (GenBank accession MN908947) and a similarity of ≥99% was considered positive for SARS-CoV-2. Early specimens were also sent to Canada's National Microbiology Laboratory (NML) for secondary confirmation and supplementary testing using various NML-developed molecular-based assays.

Next, a laboratory developed test (LDT) rRT-PCR was used for detection of the envelope (E) gene and/or SARS-CoV-2 RNA-dependent RNA polymerase (RdRp) gene using simplex or duplex methods. Duplex rRT-PCR reactions combined the primers and probe for E-gene or RdRp gene with the internal control human RNase P (RNP), **S2 Table**. Each 10 µl reaction contained 5 µl of eluted RNA, 2.5 µl of TaqPath™ 1-Step Multiplex Master Mix (Applied Biosystems), 0.4 µM each of forward and reverse primers and 0.2 µM of the labeled probe. Amplification and detection were performed using an ABI QuantStudio 5 Real-Time PCR System under the following conditions: 2 min at 25°C (uracil-DNA N-glycosylase incubation), 10 min

at 53°C (reverse transcription), 95°C (polymerase activation) for 2 min, and 45 cycles of denaturation at 95°C for 3 sec and annealing/extension at 60°C (58°C for RdRp simplex) for 30 sec. Results of the LDT rRT-PCR were based on cycle threshold (C_t) values as follows: a $C_t \leq 38$ was categorized as detected (positive), 38.1 to 39.9 was indeterminate, and a $C_t \geq 40$ was not detected (negative). Valid results required a positive result for the RNP gene internal control. The testing algorithm initially required detection of both targets for laboratory confirmation. At first E-gene and RdRp were tested in simplex reactions on the same plate. After a period, with an increase in testing volumes and percent positivity, specimens were tested first for the E-gene target due to its greater sensitivity and at the end of the day in batches by RdRp for confirmation of E-gene-positive results. This was later revised to single target detection for COVID-19 diagnosis using the E-gene target.

As of April 3, 2020 an additional rRT-PCR assay using the cobas® 8800 (Roche) was brought online and used for detection of the SARS-CoV-2. The cobas® 8800 is an all-in-one system, which includes rRT-PCR detection of the E-gene and ORF1ab. Testing was performed according to manufacturer's instructions. Specimens tested in the cobas® SARS-CoV-2 rRT-PCR assay were reported as detected or not detected; the manufacturer does not include an indeterminate range. Although the C_t value is obtainable from the instrument, the maximum number of cycles of PCR amplification used in the assay is proprietary and not provided in the kit insert and documentation.

An indeterminate result on a real-time PCR assay was defined as a late amplification signal in an rRT-PCR reaction at a predetermined high C_t value range, which is delineated by the testing laboratory at the time of validation of a LDT, or by the manufacturer for commercial assays. This may be due to low viral target quantity in the clinical specimen approaching the limit of detection of the assay or alternatively, in rare cases, may represent nonspecific reactivity (false signal) in the specimen. When clinically relevant, repeat testing is recommended.

An uninterpretable result was classified as invalid, and reflected a failure in one or more of the test's internal controls. This may be due to any number of issues, such as the presence of PCR inhibitors or poor extraction due to a highly viscous specimen. Where it was not possible to repeat the test and obtain a valid result, the invalid result was reported as such.

S2 Table. Primer and probe sequences[2] for detection of SARS-CoV-2 and the human RNAse P gene as an internal control[3], using rRT-PCR.

Primers and Probes	Sequence (5' → 3')
SARS-CoV-2 Detection	
Envelope (E) gene	
E_Sarbeco_F1	ACAGGTACGTTAATAGTTAATAGCGT
E_Sarbeco_R2	ATATTGCAGCAGTACGCACACA
E_Sarbeco_P1	FAM-ACACTAGCCATCCTTACTGCGCTTCG-BQH1
RNA-dependent RNA polymerase (RdRp)	
RdRp_SARSr-F2	GTGARATGGTCATGTGTGGCGG
RdRp_SARSr-R1	CARATGTTAAASACACTATTAGCATA
RdRp_SARSr-P2	FAM-CAGGTGGAACCTCATCAGGAGATGC-QSY
Human RNAse P (Internal Control)	
RNAse P gene	
RNase P-F	AGATTTGGACCTGCGAGCG
RNase P-R	GAGCGGCTGTCTCCACAAGT
RNP_JUN-QSY	JUN-TTCTGACCTGAAGGCTCTGCGCG-QSY

Statistical Analysis

We measured correlations between C_t values of the E-gene and RdRp targets (LDT), and the E-gene and ORF1ab (Roche) using Pearson correlation coefficients. For C_t comparisons of dual targets within the same assay, the C_t for negative results was set to 40 to not bias single target positive results.

Supplementary Reference

1. Corman VM, Müller MA, Costabel U, Timm J, Binger T, Meyer B, et al. Assays for laboratory confirmation of novel human coronavirus (hCoV-EMC) infections. Euro Surveill. 2012 Dec 6;17(49).
2. Corman VM, Landt O, Kaiser M, Molenkamp R, Meijer A, Chu DK, et al. Detection of 2019 novel coronavirus (2019-nCoV) by real-time RT-PCR. Eurosurveillance [Internet]. 2020 Jan 23 [cited 2020 Apr 21];25(3). Available from: https://www.eurosurveillance.org/content/10.2807/1560-7917.ES.2020.25.3.2000045
3. Luo W, Yang H, Rathbun K, Pau CP, Ou CY. 2005. Detection of human immunodeficiency virus type 1 DNA in dried blood spots by a duplex real-time PCR assay. J Clin Microbiol 43:1851–1857. Available from: https://jcm.asm.org/content/jcm/55/7/2035.full.pdf

A.I.II. Supplementary Test Performance Characteristics

The majority (99.0%, *n*=86,071) of specimens were tested with a single assay, the largest number of which were tested using PHO's LDT rRT-PCR assay (*n*=56,928) or the Roche platform (*n*=29,141), **S3 Table**.

S3 Table. Number of specimens tested by a single assay and with an additional assay(s): end-point RdRp gene PCR with Sanger sequencing (End Point), referred testing to the National Microbiology Laboratory (Referred), rRT-PCR laboratory developed test (LDT), rRT-PCR Roche cobas® 8800 (Roche), *n*=86,942.

	Initial Test			
Additional Test(s)	End-Point	Referred	Lab Developed	Roche
None	1	1	56,928	29,141
+Lab Developed	45	410	–	23
+Referred	370	–	0	0
+Lab Developed +Reference	23	0	0	0

Of the 871 specimens that were tested using multiple platforms, 24 (2.8%) specimens had an invalid or indeterminate result for one of the tests. Concordant results were observed for 831 (95.4%) specimens tested using multiple assays. Of the 23 specimens with discordant results, 19 were related to negative end-point PCR results, and where rRT-PCR had also been performed a median of C_t 36.2 (IQR 34.4–36.9) was observed. Details of the discordant results can be found in **S4 Table**.

S4 Table. Number of specimens by discordant result where tested by end-point RdRp PCR with Sanger sequencing (End-Point), referred testing to the National Microbiology Laboratory (Referred) (*n*=23), and of these 17 were additionally tested by rRT-PCR laboratory developed test (LDT).

End-Point	Referred	Lab Developed, Median C_t (IQR)	No. Specimens
Negative	Negative	Indeterminate, 39.5	1
Negative	Negative	Positive, 35.7 (34.3–37.2)	6
Negative	Indeterminate	Positive, 36.2 (35.4–36.8)	5
Negative	Positive	Positive 32.2 (29.8–34.5)	2
Negative	Positive	–	5
Indeterminate	Negative	Positive, 36.1	1
Positive	Negative	Positive, 34.4	1
Positive	Negative	–	1
Positive	Positive	Negative	1

Abbreviations: Ct, cycle threshold; IQR, interquartile range.

rRT-PCR assays and targets

To explore the performance of the different rRT-PCR targets, we compared test results using one or both targets across different assays. Of the 57,429 specimens tested by LDT, 3,717 (6.5%) were positive for either E-gene or RdRp-gene (**S5 Table**) – the majority of which was E-gene alone (78.1%). Where both targets were tested for a specimen (*n*=12,592), 1,077 (8.6%) were positive for both E- and RdRp-genes, 94 (0.7%) were E-gene positive only, and two specimens were indeterminate for the E-gene with one negative and one positive for RdRp. Of the 29,164 specimens tested using the Roche platform, 3,788 (13.0%) were positive for E-gene and/or ORF1ab gene, 3,507 (12.0%) were positive for both targets, 225 (0.8%) were positive for E-gene only, and 56 (0.2%) were positive for ORF1ab only. There were 7 (0.02%) specimens with invalid results which subsequently tested negative using the LDT assay.

S5 Table. Number of specimens by rRT-PCR assay result for laboratory developed test (LDT) envelope (E-gene) and RdRp targets and Roche envelope (E-gene) and ORF1ab target.

Assay Results	E-gene Results		
Second Target	Positive	Negative	Indeterminate
LDT[a]			
RdRp results			
Positive	1,077	0	1
Negative	94	11,419	1
Indeterminate	0	0	0
Not done	2,546	42,270	19
Roche[b]			
ORF1ab results			
Positive	3,507	56	–
Negative	225	25,369	–

Abbreviations: IQR, interquartile range; Max, maximum; Min, minimum.
[a]*Invalid results, n=2.*
[b]*Invalid results, n=7.*

We compared C_t values (where available) for each specimen tested using multiple targets on the same platform, which revealed highly correlated C_t values between targets (LDT R^2=0.91; Roche R^2=0.98), **S2 Fig**. However, we noted that when comparing results of the two targets tested for the same specimen the LDT E-gene C_t was lower than the RdRp-gene C_t for 896 (77.4%) of 1,158 specimens (**S6 Table**), with a median E-gene C_t of 22.4 (IQR 18.3–27.0), and a median RdRp C_t of 23.2 (IQR 19.5–27.5), p<0.001. In contrast, the Roche E-gene C_t was higher than the ORF1ab C_t for 3,339 (88.6%) of 3,768 specimens (**S6 Table**), with a median E-gene C_t of 26.7 (IQR 21.0–33.7) and a median ORF1ab C_t of 26.2 (IQR 20.6–31.9), p<0.001. Although the largest differences in C_t values for the two the targets on the same platform was up to 16.4 and 17.1 for the LDT and Roche platforms, respectively, it should be noted that the median differences were less than two C_t. While the median C_t for the E-gene on the Roche platform was slightly higher compared to ORF1ab, it is a more sensitive target as more specimens were E-positive/ORF1ab-negative (*n*=225) than ORF1ab-positive/E-negative (*n*=56), **S5 Table**.

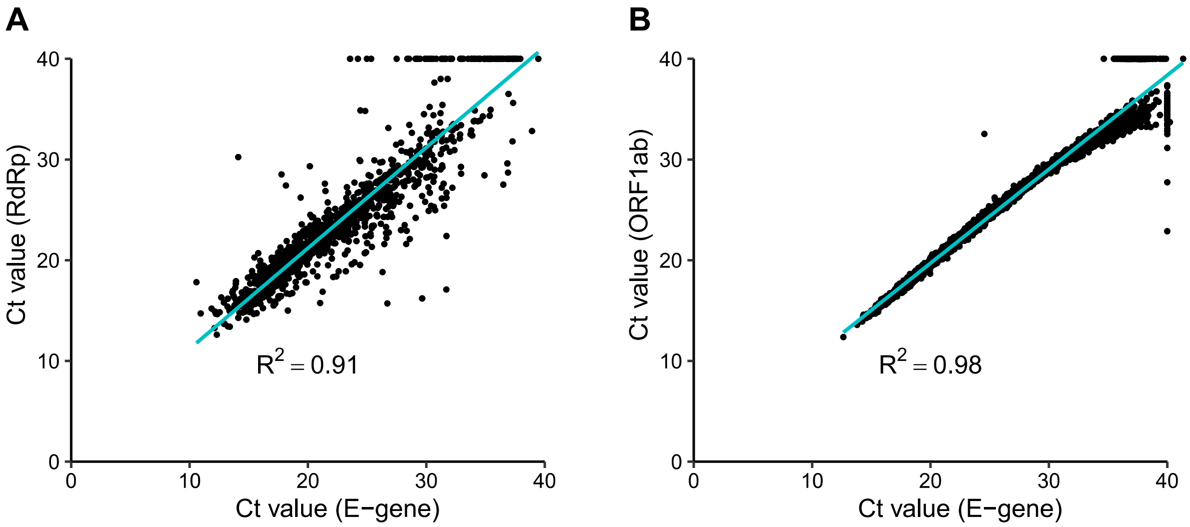

S2 Fig. Scatter plot of cycle threshold (C_t) values for tests with multiple targets. **(A)** Laboratory developed test targets, envelope gene (E-gene) and RdRp, and **(B)** Roche targets, envelope gene (E-gene) and ORF1ab. C_t values were set to 40 for a negative test result where the other target was positive to not bias the results towards those where both targets were positive.

S6 Table. Relative difference in cycle threshold (C_t) for specimens tested using rRT-PCR for both the envelope (E-gene) and RdRp targets by LDT (n=1,158), or tested for both the envelope (E-gene) and ORF1ab targets by the Roche assay (n=3,768). E-gene C_t was categorized as higher or lower than the second target C_t. C_t values were set to 40 for a negative test result where the other target was positive to not bias the results towards those where both targets were positive.

		C_t value Relative Difference	
Category	*n* (%)	Median (IQR)	Min–Max
LDT			
E-gene C_t lower than RdRp C_t	896 (77.4)	1.5 (0.8–2.5)	0.01–16.44
E-gene C_t higher than RdRp C_t	253 (21.8)	1.1 (0.4–2.4)	0.03–14.60
Identical C_t	9 (0.8)	0.0 (0.0–0.0)	0.00–0.00
Roche			
E-gene C_t lower than ORF1ab C_t	413 (11.0)	1.5 (0.1–2.5)	0.01–8.00
E-gene C_t higher than ORF1ab C_t	3,339 (88.6)	0.7 (0.3–1.5)	0.01–17.12
Identical C_t	16 (0.4)	0.0 (0.0–0.0)	0.00–0.00

Abbreviations: IQR, interquartile range; Max, maximum; Min, minimum.

A.I.III. Supplementary Molecular-based Testing Summary

Examining the different targets and assays used over the first few months of the COVID-19 pandemic showed good correlation between targets within an assay; however, we found that percent positivity and median C_t varied across different assays. This can largely be explained by a bias in assays used based on the swab manufacturer (e.g. Roche swabs used with the cobas® 8800 platform are distributed by PHO largely to hospitals), and the timeline of these assays coming online in the context of the prioritized population for testing and case rate at the time.

SII APPENDIX. Specimen-based Testing Supplementary

S7 Table. Summary for the number of specimens per individual received by Public Health Ontario, January 11, 2020 – April 22, 2020.

Number of Submitted Specimens	Number of Individuals (%)
1	74,779 (93.1)
2	4,837 (6.0)
3	568 (0.7)
4	126 (0.2)
5+	44 (0.1)

S8 Table. Number of same-day multi-specimen testing episodes categorized by SARS-CoV-2 molecular-based[a] test result and specimen types.

	Number of Testing Episodes by Results		
Specimen Types[b]	All Positive (*n*=105)	All Negative (*n*=2,882)	Positive and Negative or Other[c] Discordant (*n*=37)
Exclusively Upper Respiratory			
NPS only	23	311	12
NPS + TS	68	2,396	23[d]
NPS + Other		2	
TS only	1	8	
TS + Other		17	
Other only		2	
Exclusively Lower Respiratory			
BAL only		10	
BAL + Other		2	
Sputum only		1	
Other only		6	
Upper and Lower Respiratory			
NPS + BAL		3	
NPS + Sputum	3	28	1
NPS + TS + Sputum		28	
NPS + TS + BAL		1	
NPS + TS + Other LResp	1	1	
NPS + TS + Sputum + Other UResp		1	
NPS + Other LResp + Other UResp		1	
NPS + Other LResp		9	
TS + Sputum	1	2	
Sputum + Other UResp		1	
Non-Respiratory			
Non-Respiratory only		1	
NPS + TS + Non-Respiratory		1	
Not Specified			
NPS + TS + Not Specified	2	3	
NPS + TS + Other UResp + Not Specified			1
NPS + TS + Other LResp + Not Specified	1		
NPS + TS + BAL + Not Specified		1	
NPS + Not Specified	2	13	
TS + Not Specified	1	8	
Sputum + Not Specified		1	
Not Specified only	2	24	

Abbreviations: BAL, bronchoalveolar lavage; LResp, lower respiratory; NPS, nasopharyngeal swab, TS, throat swab, UResp, upper respiratory.

[a]*Molecular-based testing included one or more of the following: end-point PCR + Sanger sequencing of the RNA-dependent RNA polymerase (RdRp) gene, rRT-PCR to detect the envelope (E) gene, RdRp gene and/or ORF1ab.*

[b]*Specimen category "other" consists of specimens such as oral swab, saliva, tracheal, lung tissue.*

[c]*Category includes NPS-positive/TS-indeterminate result (n=1), and NPS-negative/NPS-indeterminate result (n=1).*

[d]*Comprised of 17 NPS-positive/TS-negative, 6 NPS-negative/TS-positive and 1 NPS-positive/TS-indeterminate.*

S9 Table. Number and proportion of individuals by symptom status at time of testing, for those initially testing negative and on subsequent test was either positive ≤7 days following initial negative result, or positive >7 days following initial negative.

	Subsequent Positive Test[a] *n* (%)[b]							
Initial Negative Test[a]	Symptoms (subsequent positive ≤ 7 days)				Symptoms (subsequent positive > 7 days)			
	Yes	No	Not Stated	Total	Yes	No	Not Stated	Total
Symptoms								
Yes	39 (69.6)	0 (0.0)	17 (30.4)	56 (50.9)	63 (59.4)	3 (2.8)	40 (37.7)	106 (73.1)
No	5 (83.3)	0 (0.0)	1 (16.7)	6 (5.5)	5 (83.3)	1 (16.7)	0 (0.0)	6 (4.1)
Not Stated	30 (62.5)	1 (2.1)	17 (35.4)	48 (43.6)	22 (66.7)	1 (3.0)	10 (30.3)	33 (22.8)

[a]*Molecular-based testing included one or more of the following: end-point PCR + Sanger sequencing of the RNA-dependent RNA polymerase (RdRp) gene, rRT-PCR to detect the envelope (E) gene, RdRp gene and/or ORF1ab.*
[b]*Percentages have been rounded and may not total 100%.*

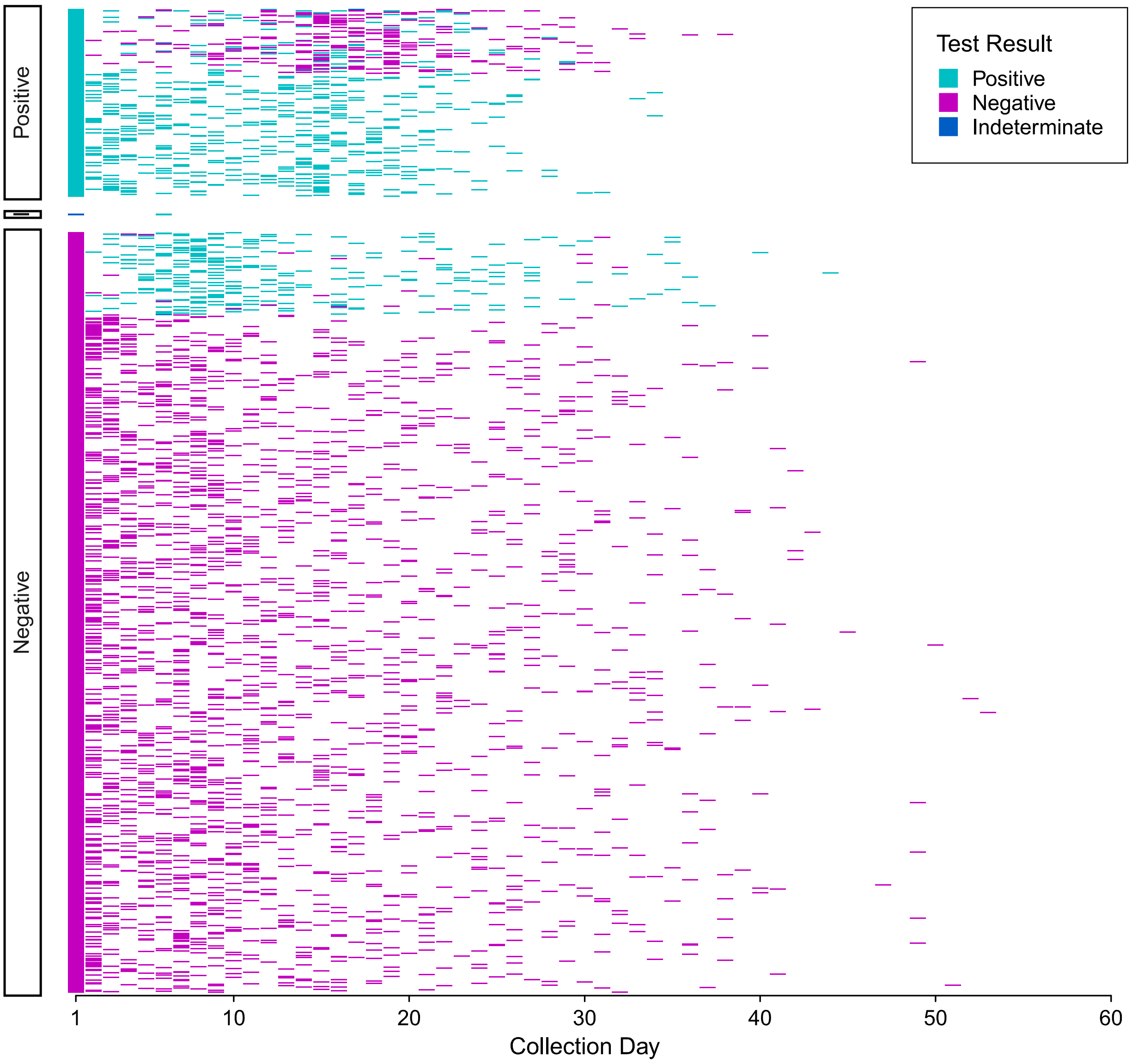

S3 Fig. Molecular-based test results for individuals with specimens collected on more than one day and submitted for testing to Public Health Ontario (*n*=2,943). Divided by initial (day 1) test result, positive, indeterminate (I) or negative, serial test results for each individual are represented horizontally across collection days. Any positive result for an individual with multiple specimens collected on the same-day is displayed as positive, and indeterminate where there was an indeterminate result and no positive. Testing included one or more of the following: end-point PCR + Sanger sequencing of the RNA-dependent RNA polymerase (RdRp) gene, rRT-PCR to detect the envelope (E) gene, RdRp gene and/or ORF1ab.
